# Supplementary material for: Exploring How Intent of Self-Harm Affects Trainee Healthcare Practitioners’ Views, Attitudes and Clinical Decision-Making in Northwest England: A Qualitative Study
Source: Int J Environ Res Public Health. 2025 Oct 14;22(10):1563. doi: 10.3390/ijerph22101563 (PMC12563878; doi:10.3390/ijerph22101563)
Supplement: Supplementary file 1 [file ijerph-22-01563-s001.zip › ijerph-3873467-supplementary.pdf]

**Supplementary File I: Interview Schedule**

*Please note that the interview schedule included questions for a separate study.

## Defining self-harm

- We gave you a definition of self-harm in the information sheet but how would you personally define it?

- SH can be defined as an intentional act. Do you think people who self-harm are in control? What about responsible for their action?

- Why do you think people self-harm?
  - *What is the goal or state that people who self-harm are trying to reach?*
  - *What are they trying to tell us as healthcare professionals?*

- How does it make you think and feel when talking about self-harm?
  - *What emotions/thoughts does it elicit in you?*
  - *How does it affect you as a practitioner? Working with self-harm.*
  - *Does this differ from how you view it personally?*
  - *Does this differ according to the patient's age?*

## Experiences of self-harm

- What are the challenges and difficulties healthcare professionals' face when working with individuals who self-harm?

  - *Are these challenges and difficulties the same or different according to whether the person is suicidal or not?*

## Intention of self-harm

- What do you think are the differences between people who self-harm for non-suicidal reasons compared to suicidal reasons?
  - *Professionally, is one of these things more challenging to work with than the other?*
  - *Do you feel that the reasons behind these two forms of self-harm differ? Why/Why not?*

- How much does intent of self-harm come into your mind when working directly with these patients?
    - *Does intent matter when working with these individuals?*
    - *Does intent affect the way you work with these individuals?*

- Do you think the intent behind self-harm affects the way other healthcare professionals work with patients? o *Why do you think this is?*
    - *Do you think this differs according to which type of healthcare professional you are? Why/Why not?*

- What does it feel like as a trainee healthcare practitioner to have responsibility for the care of patients who self-harms with suicidal intent?
    - *How does this differ from being responsible for the care of a patient who selfharms without suicidal intentions?*
    - Where do you view people who are actively vs passively suicidal?

## Location

- Do you think the location of self-harm (i.e. where it happens on the body) is significant?
    - *In what way?*
    - *Does it mean different things if an individual self-harms in a certain place as opposed to another?*

- Why do you think individuals self-harm in different locations?
    - *What do you think it means if self-harm is in a visible location?* o *How does it make you feel when you can see the self-harm e.g., the face?* o *Do you feel differently when the self-harm is in a concealed place to a visible place?*
    - *Do you think there is a stigma attached to people who self-harm in a more visible place? If so, where do you think this stigma is formed? (What about for intent NSSI/ suicidal intent)*
    - *What do you think it means if self-harm is in a concealed location?*
    - *Is there a reason they may switch from concealed to visible?*

## Clinical Decision-making

- How do you think your clinical decisions might differ for someone who self-harm with non-suicidal intentions compared to suicidal intentions?
  - *What recommendations for treatment would you make as a clinician depending on the patient's intent?*

- How does intent affect the way healthcare practitioners deliver care to people who self-harm?
  - *Are there any other factors which impact the way practitioners deliver care to these individuals? Tone, body language etc.*

- Do you think a categorical approach (suicidal and non-suicidal) is useful for clinical practice?
  - Why/Why not?

- How might the location of self-harm affect the way you responded clinically?
  - *What might you do differently if self-harm is in a visible location (e.g. the face)?*
  - *Would this treatment be any different to someone who has self-harmed in more private locations?*
- What might you do differently if self-harm is in a visible location (e.g. the face)?
- Would this treatment be any different to someone who has self-harmed in more private locations?
- How does location of self-harm affect the way healthcare professionals provide care to individuals who self-harm?
- Do all health care professionals have the same opinions of self-harm and location? Can you share any examples of different opinions?
- Are certain locations of self-harm perceived as riskier, or more emotive and would receive better care than the other locations? (What about for intent?)

## Training and Education

- Can you tell me about the training you have received about self-harm? o *What were the key messages?*
  - *How much do you know about intent and location of self-harm?*
  - *How was the session delivered?*
  - *Any thoughts/feelings on the teaching session and the teacher?*
  - *Anything surprising about what you learned?*

- Is there anything else you would like to mention or tell me about we have not covered today?

**Supplementary File II: Process of theme development**

Braun & Clarke's, [107] RTA entails six stages which included data familiarisation, code generation, theme generation, reviewing and defining themes and reporting the results of the analysis through conducting a write-up.

*Phase 1, familiarisation*: During transcription of the interviews, DK underwent an interpretative process of listening to the audio recording of the data and writing the transcript to become familiar with the essence of the data [106]. As suggested by Braun & Clarke, [107], DK asked themselves reflective questions when reading the transcripts and made preliminary notes of initial thoughts and interpretation of meanings.

*Phase 2, code generation:* After transcription had occurred and DK had re-read the transcripts several times, DK analysed each transcript separately by hand. DK explored the transcript to identify meaningful data excerpts which helped understand what trainee healthcare practitioners' views and understandings were about working with different forms of self-harm and how that affected their clinical decision making. DK assigned descriptive, inductive codes which were informed through referring to the study aims. An inductive approach was taken through exploring the participant's personal narrative and summarising the essence of their expressions. However, a deductive approach also came through from using psychological theory to make sense of participants' experiences. Using Microsoft Word, the codes were further refined to condense the number of codes and find recurrence in the coding. Initially, coding was conducted at a semantic level when describing attitudes towards self-harm, different types of intent and how that affected trainees' clinical decision making. Gradually codes were developed to represent latent meanings through identifying inconsistencies and mismatches in trainees' thoughts, feelings and behaviours towards self-harm, as well as the perceived responsibility for the act. Once all interviews were coded, DK reviewed the codes to ensure that highly similar codes were combined to represent a single construct.

*Phase 3, constructing themes:* Initial themes were generated through grouping similar codes into broader categories which reflected patterns of meaning across respondents. A descriptive and interpretive lens was used when developing themes. A descriptive approach was evident when participants explicitly stated their attitudes or clinical decision-making around self-harm whereas an interpretative lens was important when considering implicit

themes such as 'powerlessness' or practitioners' appraisals of controllability of the behaviour. Whilst the lead researcher developed initial themes, supervision was utilised with a clinical psychologist to define, name and consolidate themes. A hierarchical thematic structure was generated and adapted three times. Themes which contained clusters of codes which overlapped and had the same meaning (e.g. 'Decisions around treatment planning' and 'Approach to care') were merged.

*Phase 4 and 5, revising and defining themes:* Next, the themes were reviewed by referring to significant statements to ensure that they voiced trainees' views of non-suicidal and suicidal self-harm [108], had informative names and were distinct in nature.

*Phase 6, writing the report:* Lastly, DK wrote up the findings through a narrative which weaved analysis, significant statements and context together. Throughout the analysis memo writing and reflexive journaling was conducted to record DK's sense-making of the data and ensure transparency. These records provided an audit trail of how codes evolved into themes and how analytic decisions were made over time, supporting transparency and rigour.

Additional References


106. Bird, C. M. (2005). How I stopped dreading and learned to love transcription. *Qualitative inquiry*, *11*(2), 226-248. https://doi.org/10.1177/1077800404273413

107. Braun, V., & Clarke, V. (2012). Thematic analysis. In H. Cooper, P. M. Camic, D. L. Long, A. T. Panter, D. Rindskopf, & K. J. Sher (Eds.), *APA handbook of research methods in psychology, Vol. 2. Research designs: Quantitative, qualitative, neuropsychological, and biological* (pp. 57–71). American Psychological Association. https://doi.org/10.1037/13620004

108. Rizq, R., & Target, M. (2008). 'The power of being seen': An interpretative phenomenological analysis of how experienced counselling psychologists describe the meaning and significance of personal therapy in clinical practice. *British Journal of Guidance & Counselling*, *36*(2), 131-153. https://doi.org/10.1080/03069880801926418
